# Artificial Intelligence‐Based Pathology to Assist Prediction of Neoadjuvant Therapy Responses for Breast Cancer

**DOI:** 10.1002/cam4.71132

**Published:** 2025-08-05

**Authors:** Juan Ji, Fanglei Duan, Qiong Liao, Hao Wang, Shiwei Liu, Yang Liu, Zongyao Huang

**Affiliations:** ^1^ Department of Pathology, Sichuan Clinical Research Center for Cancer, Sichuan Cancer Hospital & Institute, Sichuan Cancer Center Affiliated Cancer Hospital of University of Electronic Science and Technology of China Chengdu China; ^2^ Department of Breast, Sichuan Cancer Hospital & Institute, Sichuan Cancer Center, School of Medicine University of Electronic Science and Technology of China Chengdu China

**Keywords:** artificial intelligence, breast cancer, neoadjuvant therapy, pathology, prediction

## Abstract

**Background:**

Neoadjuvant therapy (NAT) is a standard breast cancer treatment, but patient response varies significantly. Predictive markers can guide treatment decisions, yet their interpretation suffers from inter‐pathologist variability due to breast cancer's complex histology and heterogeneity. Artificial intelligence (AI) applied to image‐based omics offers potential to enhance pathological interpretation precision and consistency.

**Methods:**

This review synthesizes existing literature on the application of AI in breast cancer pathology. We specifically focused on identifying and summarizing research that utilizes diverse histopathological features—including morphological characteristics, molecular markers, gene expression profiles, and multidimensional omics data—to predict NAT response in breast cancer patients.

**Results:**

AI demonstrates significant capabilities in automatically recognizing histopathological patterns and predicting NAT efficacy. It shows promise as a tool for patient stratification in precision oncology. Research utilizing various pathological feature types (morphological, molecular, genomic, multi‐omics) for NAT response prediction is actively evolving. While AI models integrating multi‐omics features show potential, challenges remain in robustly predicting NAT outcomes.

**Conclusion:**

AI‐based pathology represents a prospective and powerful decision‐support tool for predicting breast cancer NAT response. Despite existing challenges, particularly with complex multi‐omics models, AI holds great potential to assist clinical oncologists in optimizing future cancer treatment management.

## Graphical abstract

The integration of pathological omics data through computational methods shows promise in advancing precision oncology for breast cancer. In this article, we will review evidence supporting the use of AI‐assisted pathology features (morphological, molecular, and genetic features) and multi‐dimensional profiling to predict neoadjuvant therapy response in breast cancer.

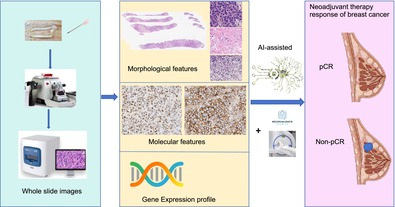

## 1 Introduction

Breast cancer is the most common malignant tumor in women worldwide [1]. Neoadjuvant therapy (NAT) can improve treatment outcomes, but patient responses vary greatly [2]. Conventional methods for evaluating NAT response, such as histopathological and biomarker assessment, have limitations in accuracy and efficiency [3].

Emerging digital pathology technologies offer a solution by enabling faster, more objective evaluation of tumor characteristics and treatment response. Digital pathology integrates pathology images, including morphology and molecular data, for computational analysis across diseases. Studies show digital histology achieves consistency with traditional glass slides and sometimes surpasses diagnostic performance [4, 5].

**FIGURE 1:**
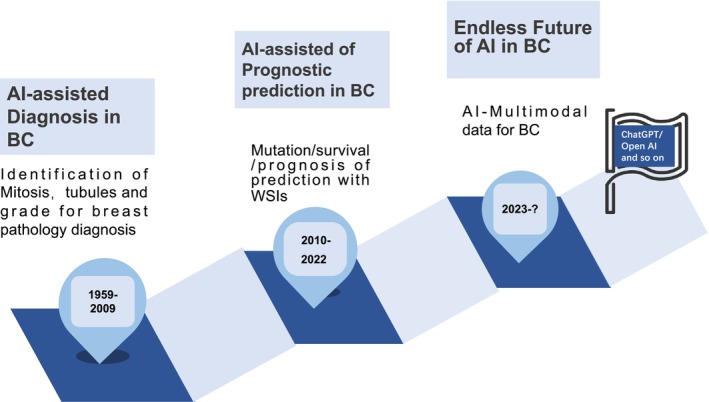
The development history of AI and machine learning in BC pathology. The application of artificial intelligence (AI) in the field of breast cancer (BC) can be divided into three stages: (1) diagnostic assistance (1959–2009) that, based on traditional machine learning, mitosis, tubular structure, and histological grade, were identified from pathological images to enhance the standardization of diagnosis. (2) Prognosis prediction (2010–2022), relying on deep learning, integrating full‐section images and clinical data, predicts survival rates, recurrence risks, and gene mutations, and promotes precise treatment. (3) Future Trends (2023–) stage, focusing on the fusion of multi‐modal data (pathology, genomics, imaging, text) and generative AI (such as ChatGPT) to achieve end‐to‐end diagnosis and treatment decision‐making.

Digital pathology emerged in the 1960s, enabling tumor characterization through quantifiable cellular attributes derived from digitized slides [6] (Figure 1). Subsequent technological advances facilitated computational analysis through visual feature extraction methodologies, including scale‐invariant feature transform and speed up robust features [7, 8]. Machine learning algorithms, such as support vector machines and random forests, established correlations between predefined image features and variables of interest. Contemporary deep learning approaches, particularly convolutional neural networks (CNNs), have revolutionized digital pathology omics analysis [9, 10]. CNNs distinguish themselves from traditional methodologies by automatically extracting relevant patterns through visualization concepts, eliminating the need for manual feature engineering. CNNs, a key component of deep learning, have transformed computer vision and digital pathology analysis.

While CNNs have existed since the 1980s, it was not until the early 2010s that algorithms were developed to leverage larger datasets and more complex computations. These deep learning techniques form the foundation of Artificial Intelligence (AI) approaches, greatly enhancing the automated assessment of digitized tumor pathology slides. AI systems can augment human capabilities by rapidly screening large volumes of samples and extracting clinically relevant insights. This promotes the development of automated diagnostic tools to alleviate pathologist workloads in the future. AI has expanded beyond conventional H&E sections, enabling the prediction of abstract categories and spatial distribution characteristics from limited image data [11, 12]. Furthermore, advanced end‐to‐end AI methods, unconstrained by predefined patterns, can integrate subtle visual cues to predict risk scores and survival outcomes for various cancer types, including hepatocellular carcinoma [13], colorectal cancer [14], and brain tumors [15]. The transformative impact of CNNs and AI on digital pathology highlights their potential to enhance diagnostic accuracy, workflow efficiency, and personalized treatment approaches.

It is clinically meaningful to predict responses to specific therapies, enabling better treatment recommendations from clinicians. Previous research across multiple cancer types has shown that AI can infer some molecular or genetic changes directly from HE‐stained histopathology slides, thereby predicting drug effects and tumor treatment responses [16, 17, 18, 19]. Breast cancer is among the tumors with the most rapid development of neoadjuvant treatment options. Consequently, numerous AI‐based pathological omics studies have evaluated neoadjuvant efficacy in breast cancer. Studies have found that morphological, molecular, and genetic features of breast cancer tissues identified by AI can predict neoadjuvant response. Additional work has constructed multi‐dimensional models integrating different pathological, radiomic, molecular, and proteomic features to predict neoadjuvant efficacy in breast cancer.

## 2 Application and Research of AI Learning Model in Breast Cancer Histological Feature Recognition

Histological feature serves as a robust determinant of survival prognosis in breast cancer patients. The Nottingham Grading System, the primary method for characterizing breast cancer histological morphology, evaluates three key elements: nuclear pleomorphism, mitotic count, and tubule formation. Each component provides crucial prognostic information, intricately linked to patient outcomes.

Recent research has applied AI models to extract morphological features from histopathology, assessing tumor phenotypes and enabling risk stratification (Table 1).

**TABLE 1:** The application of AI in breast cancer pertaining to histopathology.

Research group	Cohort size	AI technology	Specific networks	Performance	Application
Paul et al. [20]	450 H&E stains	Area morphological scale space	/	F1 score: 0.71–0.83	Automatic mitosis detection
Wahab et al. [21]	78 H&E stains	Deep learning	CNN; ResNet	F measure: 0.79	Classify mitotic and non‐mitotic nuclei
Xu et al. [22]	500 H&E stains	Deep learning	SSAE	F measure: 0.8449 AUC: 0.7883	Identify distinguishing features of nuclei
M et al. [23]	39 cases	Deep learning	Deep CNN; ReLU	*r* ^2^: 0.77	Measure nuclear area
Xing et al. [24]	30 brain tumor 51 NET 35 breast cancer	Supervised learning	Deep CNN	Brain tumor: F1 score: 0.60–0.77 NET: F1 score: 0.72–0.88 Breast cancer: F1 score: 0.46–0.78	Nucleus Segmentation
Veta et al. [25]	23 cases	Deep learning	CNN A random forests classifier	F1 score: 0.611	Mitosis detection
Amgad et al. [26]	125 cases	Deep learning	Mask R‐CNN model	AUC: 0.75–0.84	Nucleus classification and segmentation
Nateghi et al. [27]	500 WSIs	Deep learning	Deep neural networks Support Vector Machine (SVM) classifier	F1 score: 0.7381	Mitosis detection
Romo‐Bucheli et al. [28]	174 WSIs	Deep learning	DNN (deep neural network)	AUC 0.76	Oncotype DX risk categories
Janowczyk et al. [29]	1200 WSIs	Deep learning	AlexNet Network	Classification accuracy of 0.97	Distinguish of different histopathological morphology
Wang et al. [30]	1567 cases	Deep learning	DeepGrade CNN model	AUC: 0.919–0.937	Risk stratification for Nottingham histological grade
Boissin et al. [31]	896 cases	Deep learning	Deep convolutional neural network model	AUC: 0.908	Risk stratification for Nottingham histological grade
Luo et al. [32]	14,243 cases	Deep learning	DeepSurv	C‐index: 0.791	Risk stratification for overall survival
Huang et al. [33]	733 cases	Deep learning	Deep neural networks	AUC: 0.87	Risk stratification of ADH and DCIS
Albusayli et al. [34]	429 cases	Deep learning	Deep learning‐based algorithm	C‐index: 0.76	Risk stratification for disease‐specific survival
Mondol et al. [35]	249 cases	Deep learning	BioFusionNet	AUC: 0.84	Survival risk stratification for ER+ breast cancer

Abbreviations: ADH, atypical ductal hyperplasia; CNN, Convolutional Neural Network; DCIS, ductal carcinoma in situ; FGDC‐net, Feature Global Delivery Connection Network‐net; HE, ematoxylin‐eosin; NET, neuroendocrine tumor; ReLU, Rectified linear unit; SSAE, Stacked Sparse Autoencoder; WSIs, whole slides.

### 2.1 Application of AI Learning Model in Breast Cancer Nuclear Pleomorphism Feature Recognition

The “pleomorphism” of tumor cell nuclei includes irregular nuclear shapes, nuclear sizes, and changes in the number and distribution of chromatin. The presence of polymorphic nuclei is an indicator related to tumor prognosis. While the fifth edition of the WHO classification of breast tumors provides established criteria for nuclear grading, significant interobserver variability persists in the assessment of nuclear pleomorphism among pathologists [36]. With the aid of deep learning, the nuclear pleomorphism recognition model developed by Caner Mercan et al. can achieve top pathologist‐level performance in multiple experiments on regions of interest and whole‐slide images compared to other pathologists [37]. Previous studies have proven that the appearance of nuclear pleomorphism may be related to the mitotic count, and detailed analysis of the nuclear shape can help distinguish between mitotic and non‐mitotic nuclear classifications [20, 38].

The Wahab [21] study implemented a CNN‐based two‐stage analytical framework utilizing a global binary threshold method for HE image analysis: Stage I involved differentiating straightforward, typical, and challenging mitotic figures, with challenging cases undergoing specimen flipping prior to rotational augmentation to enhance detection probability, subsequently processed through the secondary classifier. The study in Xu [22] also proposed an efficient deep neural network architecture based on stacked sparse autoencoders (SSAE) specifically for recognizing nuclear atypia. The authors showed that the deep SSAE outperformed “shallow” architectures in terms of nuclear detection accuracy. Other nuclear pleomorphism studies, including M. Veta's study [23], also demonstrated that precise measurements of individual nuclear areas and area statistics, such as average nuclear area, can be directly obtained through deep CNN models, thereby bypassing the intermediate nuclear segmentation step to achieve intelligent recognition. In addition, Pro [24] and other authors proposed the use of a “shape‐preserving” automatic learning method for nuclear segmentation, in which the algorithm iteratively improves and feeds the CNN‐generated “shape” probability map into a segmentation algorithm that rejects deformation models based on selected sparse shape models and local shapes.

### 2.2 Application of AI Learning Model in Recognition of Nuclear Mitoses in Breast Cancer

AI learning models have revolutionized nuclear mitosis recognition in breast cancer pathology. These deep learning‐based models analyze high‐resolution histopathological images to detect and classify mitotic figures with increasing accuracy.

Two landmark studies, MITOS‐ICPR (2012) [39] and AMIDA13 (2013) [25], significantly advanced this field. MITOS‐ICPR established a benchmark dataset and evaluation metrics for automated mitosis detection in H&E‐stained breast cancer specimens. AMIDA13 expanded on this work, providing a larger, more diverse dataset to evaluate automated mitosis detection methods in whole‐slide images. Both studies employed machine learning algorithms, including CNNs, achieving high detection accuracy. Recent advancements include Mohamed Amgad et al.'s NuCLS dataset, comprising over 220,000 breast cancer cell nuclei, which enables faster predictions for NAT response [26]. Ramin Nateghi et al. developed a deep learning model for automatic mitosis counting in whole‐slide images (WSIs), achieving an *F*‐value of 73.81% and a weighted kappa score of 0.612 [27].

These studies demonstrate AI's potential to assist pathologists in mitotic count assessment, a crucial factor in breast cancer grading. The integration of AI‐based mitosis detection tools into clinical workflows promises to standardize breast cancer grading, reduce inter‐observer variability, and expedite diagnosis. Nevertheless, significant challenges persist in ensuring the robustness, interpretability, and clinical validation of these models, which must be addressed prior to their widespread implementation in clinical practice.

### 2.3 Application of AI Learning Model in Breast Cancer Glandular Recognition

Tubule formation and proportion are also one of the important manifestations of breast cancer histology. Despite the complexity of ductal recognition and segmentation, in the study by Romo‐Bucheli [28], the authors used a recursive neural network architecture to identify and segment ducts by detecting the inner edge of the lumen and the outer edge of the duct. Subsequently, another CNN detected and counted the nuclei between the two edges, thereby extracting a parameter called the ductal formation index. This index was then used to predict the OncotypeDX results of 174 patients, with an AUC value reaching 0.76. Another study [29] also showed that CNNs can be used to separately extract and recognize the ductal structure features of breast cancer. Although the accuracy of ductal analysis with CNNs has not yet reached the level of complete automation for clinical use without manual evaluation, it is being addressed with deeper neural network architectures and transfer learning strategies, and it has been incorporated into more comprehensive tumor detection and staging frameworks [30].

### 2.4 Applications of AI Learning Models in Risk Stratification Based on Histopathology

While histological grading systems demonstrate robust interobserver concordance for high‐grade neoplasms (NHG 3), persistent diagnostic discordance persists in differentiating NHG 1 and NHG 2 categories [40, 41]. This variability stems from inherent limitations in subjective pathological interpretation and methodological heterogeneity across diagnostic platforms. Such inconsistencies critically impair prognostic stratification and therapeutic decision‐making for intermediate‐grade malignancies.

Emerging AI‐driven computational pathology approaches address these limitations through standardized quantitative analysis. The DeepGrade framework, a validated deep learning model utilizing whole‐slide images, reclassifies histologically ambiguous grade 2 tumors into molecularly distinct high‐risk (grade 3‐equivalent) and low‐risk (grade 1‐equivalent) subgroups [31, 42]. This computational stratification enhances prognostic precision and informs targeted therapeutic strategies beyond conventional grading paradigms. In recent years, numerous studies have employed AI to analyze breast cancer histological morphology, facilitating more refined risk stratification across diverse patient populations and tumor subtypes.

Luo et al. [32] developed a deep learning‐based clinical risk stratification model for overall survival in young women with breast cancer. Their model integrated histological features with clinical data, achieving superior predictive performance compared to traditional prognostic tools. Huang et al. proposed a deep learning model to improve histological grading and predict upstaging of atypical ductal hyperplasia and ductal carcinoma in situ on breast biopsies [33]. Their approach demonstrated high accuracy in distinguishing between low‐ and high‐grade lesions, potentially reducing unnecessary surgical interventions. Using routine digital histopathology images, Sharma et al. validated an AI‐based solution for breast cancer risk stratification [43]. While in triple‐negative breast cancer (TNBC), Albusayli et al. utilized AI‐based digital scores of stromal tumor‐infiltrating lymphocytes and tumor‐associated stroma to predict disease‐specific survival [34]. Their method showed improved prognostic value over manual assessment, highlighting the potential of AI in quantifying complex histological features. Mondol et al. introduced BioFusionNet, a deep learning‐based model for survival risk stratification in ER+ breast cancer [35]. By integrating multifeature and multimodal data, including histopathological images and genomic information, their approach achieved superior performance in predicting long‐term survival outcomes.

These studies demonstrate the increasing efficacy of AI in assessing histological morphology for breast cancer risk stratification across diverse populations, underscoring its potential for clinical integration.

## 3 Application of AI for Predicting the Efficacy of NAT for Breast Cancer Based on Pathological Features

**FIGURE 2:**
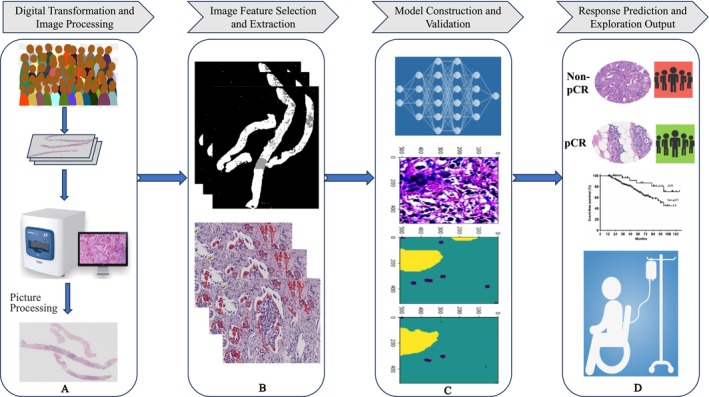
General workflow of AI‐based pathology for response prediction in breast cancer. (A) Digital Transformation Phase: Histopathological specimen digitization through whole‐slide imaging followed by standardized preprocessing (tissue segmentation, color normalization, and artifact removal). (B) Feature Engineering Stage: Automated extraction of quantitative morphometric features (nuclear pleomorphism, stromal architecture) coupled with deep learning‐based feature selection for dimensionality reduction. (C) Predictive Modeling Framework: Development of ensemble AI architectures integrating convolutional neural networks with clinical covariates, validated through multicenter external cohorts using bootstrap resampling. (D) Clinical Translation Output: Probabilistic prediction maps stratifying pCR possibility with performance metrics.

The AI prediction of the efficacy of NAT for breast cancer based on pathological histology usually includes the following parts (Figure 2): (a) digital transformation and preliminary image processing of pathological images; (b) marking, extraction, and selection of input image features; (c) model construction and validation; (d) pCR prediction and exploration of outputs.

Integrating digital pathology and AI has significantly advanced breast cancer diagnosis and treatment by enabling individualized evaluation, especially of NAT efficacy prior to systemic drugs. Beyond traditional H&E staining, pathomics encompasses molecular markers associated with drug response including ER, PR, HER2, Ki67, and PD‐L1, as well as related genomic and proteomic datasets that reflect tumor sensitivity to existing therapies. By merging these diverse pathomic features with sophisticated AI approaches, researchers can predict the efficacy of neoadjuvant regimens for breast cancers. This computational pathomics holds promise for optimizing personalized care through improved response prognostication and treatment selection (Table 2).

**TABLE 2:** The application of AI in breast cancer NAT response prediction.

Research group	Cohort size	AI technology	Specific networks	Performance	Genomic subtypes
Xu et al. [44]	68 cases	Deep learning	Unet++	AUC: 0.908	NS
Zeng et al. [45]	440 cases	Deep learning	SVM classifier	AUC: 0.71–0.79	All types
Fisher et al. [46]	85 cases	Machine learning	Machine learning pipeline	Accuracy: 0.835	TNBC
Li et al. [47]	540 cases	Deep learning	CNN; stochastic gradient descent (SGD)	Accuracy: 0.853 F1 score: 0.503 AUC: 0.822	All types
Saednia et al. [48]	207 cases	Hierarchical self‐attention‐guided deep learning	Customized ViT network CoAtNet model	F1‐score: 0.90 AUC: 0.89	All types
Saednia et al. [49]	149 cases	Machine learning	U‐Net model	AUC: 0.90 Sensitivity: 85% Specificity: 82%	All types
Krishnamurthy et al. [50]	243 cases	Deep Convolutional Neural Network–Based AI Tool	CNN	AUC: 0.75	TNBC
Li et al. [51]	1035 cases	Deep learning	CNN	AUC: 0.673–0.745	All types
Hacking et al. [52]	120 cases	Multi‐omic machine learning	Tree‐based Pipeline Optimization Tool (TPOT)	AUC: 0.62–0.78	TNBC
Basavanhally et al. [53]	12 cases	Unsupervised Varma‐Zisserman texton‐based classification scheme	Support vector machine (SVM) clustering iterated conditional modes (ICMs) algorithm	Accuracy: 0.89	HER2‐positive
Saltz et al. [54]	5202 WSIs	Deep‐learning	CNN	AUC: 0.9544	NS
Amgad et al. [55]	120 WSIs	Fully Convolutional model	VGG16‐FCN8 and FC‐DensNet103	AUC: 0.78–0.90	TNBC
Lu et al. [56]	1000 WSIs	Deep‐learning–based pipeline	U‐net–based neural network; Resnet18 model	Recall, 0.9536; Precision, 0.901; F1score = 0.9266	ER positive ER Negative TNBC
Negahbani et al. [57]	2357 WSIs	Deep learning	DNNs; PathoNet; U‐net	F1 score: 0.7928	NS
Le et al. [58]	1090 cases	Deep learning	CNN; 34‐layer ResNet, 16‐layer VGG, Inception v4	F1 score: 0.893; Accuracy: 0.89 AUC = 0.950	NS
Makhlouf et al. [59]	2231 cases	Deep learning	CNNs	F1 score: 0.81–0.92	Luminal BC
Fanucci et al. [60]	113 cases	Deep learning	CNN	AUC: 0.627–0.709	HER‐2‐negative BC
Ma D et al. [61]	60 cases	Deep learning	KNN	AUC = 0.83	HER2‐positive breast cancer
Ogier et al. [62]	529 cases	Machine learning	Collaborative FL	AUC: 0.72	TNBC
Aswolinskiy et al. [63]	926 patients	Deep learning	CNN	AUC: 0.88	TNBC Luminal B
Shen et al. [64]	310 patients	Deep learning	SVMs; CNNs; ResNet‐50	AUC: 0.905–0.967 Accuracy: 95.15%	All types
Li et al. [65]	874 cases	Deep learning	Deep learning	AUC: 0.72–0.82	All types
Shamai et al. [66]	3376 cases	Deep learning	CNN	AUC: 0.91–0.93	ER Positive ER Negative
Farahmand et al. [67]	188 WSIs	Deep learning	CNN	AUC: 0.90	HER2 Positive
Wang et al. [68]	109 IHC stains	Deep learning	AI‐assisted model	Accuracy: 0.96	NS
Huang et al. [69]	62 cases	Machine learning	IMPRESS	AUC: 0.897 AUC: 0.767	HER2‐positive TNBC
Sammut et al. [70]	168 cases	Machine learning	Multi‐step predictor pipeline.	AUC: 0.87	ER‐positive ER‐negative HER2‐positive HER2‐negative
Mao Net al [71].	1004 cases	Deep learning	Automated pipeline system	AUC = 0.909	NS

Abbreviations: All types include: Luminal BC, HER2‐positive BC and TNBC; CNNs, sconvolutional neural networks; DNNs, deep neural networks; FL, federated learning; IMPRESS, IMage‐based Pathological REgistration and Segmentation Statistics; KNN, k‐nearest neighbor algorithm; NS, NO specific; SGD, stochastic gradient descent; SVM, Support vector machine; TNBC, triple negative breast cancer.

### 3.1 Construction of NAT Prediction Model Bassed on Histopathological Features

Breast cancer exhibits a wide spectrum of histopathological morphologies. Recent studies [44, 45, 46] have utilized AI models to analyze diverse histological features, including epithelial and stromal characteristics, for predicting NAT efficacy in different subtypes.

#### 3.1.1 Construction of Neoadjuvant Treatment Prediction Model Based on AI‐Assisted Breast Cancer Epithelial Features

Our previous study found that tumors with higher histological grades were more likely to achieve pathologic complete response (pCR) following NAT versus lower‐grade lesions [72]. Collins and Pastorell also corroborated the association between breast cancer histological grade and neoadjuvant efficacy [73, 74]. Numerous investigations have validated deep learning for precise histological subtyping of breast cancer [27]. Can deep learning also predict neoadjuvant response by extracting visible and subtle epithelial features from digitized pathology images? Studies by Li et al., Saednia et al., and others addressed this question [47, 48, 49]. Using CNNs or self‐attention models applied to pre‐treatment breast tumor slides, the researchers predicted neoadjuvant efficacy with AUCs up to 0.85–0.90. In early‐stage TNBC, another group developed and validated a histopathology‐based deep learning model for forecasting neoadjuvant response, achieving an AUC of 0.88 [50]. The study also revealed significant correlations between molecular subtypes and pCR scores in predicting NAC response rates, with HER2+ and HR‐ subtypes showing significantly higher pCR scores [47]. Furthermore, histological analysis demonstrated that morphological characteristics observable in HE images may serve as biomarkers for molecular subtyping and further to NAC efficacy prediction [75].

This computational approach demonstrates clinical potential in optimizing personalized breast cancer management, particularly for refractory subtypes, by providing standardized, rapid, and cost‐effective assessments that circumvent inter‐pathologist variability. Further histopathological characterization may elucidate critical insights into tumor biology and phenotypic drug susceptibility.

#### 3.1.2 Construction of a Neoadjuvant Treatment Prediction Model Based on AI‐Assisted Tumor Associated Stroma Features

Hematoxylin and eosin (HE) stained pathological images contain complex, rich information reflecting molecular processes and disease progression. In addition to epithelial tumor cell features enabling histological grading, they also provide abundant stromal traits. The TME, including the associated extracellular matrix, constitutes a suitable environment for cancer growth, progression, and metastasis. The histological stromal phenotype reflects collective effects of potential biological changes.

Previous research demonstrates different tumor stromal subtypes associate closely with the efficacy of NAT, acting as predictors of treatment response in breast cancer [76, 77, 78]. Stromal analysis from HE slides holds potential for personalized prognostication and therapy selection.

However, the inherent complexity and heterogeneity of breast cancer pathology images hamper manual assessment and information gleaning. The rapidly developing AI showed promise to address this barrier. In the research of Nehal M. Atallah [79], AI is used to evaluate the stroma‐to‐tumor ratio and spatial matrix cell distribution in 1968 breast cancer cases. The results show that breast cancer patients with high S:TR ratios correlated with favorable prognosis, whereas uneven ratios portended worse outcomes. In a multicenter study, Li et al. constructed an end‐to‐end CNN model predicting neoadjuvant response that identified tumor‐associated stroma subtypes including fibrous, collagenous, and lymphocyte‐rich infiltrates [51]. Lymphocyte‐rich stroma associated with improved response (AUC 0.80). Additionally, Hacking et al. computationally characterized distinct stromal compositions in TNBC, finding collagen‐rich stroma predicted poorer pathological complete response (AUC 0.77) relative to mixed mucinous stroma [52]. High collagen density in the TME restricts drug diffusion through increased tissue stiffness while activating cancer‐associated fibroblasts (CAFs) to secrete immunosuppressive factors like TGF‐β, collectively diminishing therapeutic efficacy [80]. AI models can predict treatment response by quantifying spatial features (e.g., matrix ratio, collagen component) that correlate with intratumoral drug distribution dynamics.

Collectively, these findings illustrate AI's potential for elucidating subtle morphologic stromal features' prognostic and predictive value in breast cancer, addressing limitations of manual evaluation through computational pathology. Further research may optimize individualized management.

### 3.2 AI‐Assisted Intelligent Evaluation of Tumor Infiltrating Lymphocytes and Its Application in Predicting NAT

Tumor infiltrating lymphocytes (TILs) are considered to be a key player in the TME of breast cancer. Elevated levels of TILs in HER2‐positive and TNBC have been associated with enhanced chemosensitivity through CD8+ T cell‐mediated cytotoxicity and IFN‐γ signaling pathways [81, 82]. Despite the 2023 CSCO guidelines endorsing the assessment of TILs, the clinical implementation remains constrained by tumor heterogeneity and diagnostic variability.

AI has the potential to overcome these limitations through standardized quantification. Basavanhally et al. pioneered the automated detection of TILs in HER2‐positive cases, achieving an accuracy of over 90% and establishing the technical paradigm [53]. Subsequent deep learning models have achieved precise lymphocyte mapping on H&E slides by recognizing spatial infiltration patterns, such as dense peritumoral clusters versus diffuse stromal distributions [54, 55, 56, 57, 58, 59]. This computational precision has enabled deeper biological insights, with studies demonstrating that high TIL scores (AUC = 0.804) correlate with the formation of tertiary lymphoid structures, suggesting sustained immune activation crucial for treatment response [51]. Similarly, combining digital pathology with spatial omics technology, a CNN classifier linked the spatial density of TILs in HER2‐negative cases to TNF‐α pathway enrichment, explaining its predictive capacity for pathological complete response [60]. In another study of HER2‐positive breast cancer patients treated with the new neoadjuvant ADC drug SHR‐A1811, Ma D et al. [61] constructed a predictive model based on clinical pathological features and pathological images. This model performed well in the SHR‐A1811 training set (AUC = 0.95), test set (AUC = 0.86), and real‐world ADC validation set (AUC = 0.83). Moreover, the study found that a high proportion of immune cell infiltration, especially cytotoxic T cells, was associated with a better treatment response. Further research revealed that the high aggregation of immune cells might be related to the high expression of downstream genes of ESR1, such as PGR and CCND1, which further affected the therapeutic efficacy of SHR‐A1811.

Additionally, federated multi‐instance learning methods augmented sample sizes for further evaluating the relationship between TILs and forecasting neoadjuvant efficacy [62]. Aswolinskiy's neural network quantified four biologically grounded indicators: (1) Lymphocyte‐tumor ratio (immune effector capacity), (2) Inflamed tumor ratio (pro‐inflammatory niche), (3) Mitotic rate (proliferative vulnerability), achieving a record AUC of 0.88 in TNBC cases [63]. Multicomponent analyses further reveal the complex tumor‐stroma interplay. Shen and Krishnamurthy's CNN models, integrating epithelial glandular disruption with stromal desmoplasia, achieved 95.15% accuracy in TNBC prediction [64, 76]. This aligns with the biological evidence that collagen remodeling facilitates drug penetration, while epithelial dedifferentiation increases therapeutic susceptibility [65]. Such AI‐derived composite biomarkers transcend the isolated assessment of TILs, capturing the dynamic tumor‐immune‐stroma axis governing the response to neoadjuvant therapy.

### 3.3 Application of AI Models Based on Molecular Detection in Predicting NAT for Breast Cancer

The systemic management of breast cancer relies on molecular subtyping based on hormone receptor and HER2 status. Subtypes with hormone receptor negativity and HER2 positivity exhibit higher pCR rates to NAT, potentially due to HER2 signaling activation and hypoxia‐inducible factor 1 (HIF‐1) pathway upregulation, suggesting intensified anti‐HER2‐targeted therapy may enhance efficacy [83, 84]. Elevated Ki67 also correlates with poorer prognosis yet increased chemotherapy sensitivity [85].

Conventional biomarker assessment is resource‐intensive, but artificial intelligence (AI) can infer molecular profiles and predict therapy responses directly from routine histology [66, 67, 86, 87]. For example, Farahmand's CNN model classifier achieved an AUC of 0.80 in the five‐fold cross‐validation, accurately predicting the HER2 status of breast cancer patients and the response to trastuzumab treatment [67].

In addition to HER2‐positive breast cancer, in the study of Savitri Krishnamurthy and others [50] deep learning algorithms were used to evaluate the response to NAT in 165 cases of TNBC, and the AUC reached 0.75, suggesting that it can effectively predict the treatment response of this highly invasive type of breast cancer, provide treatment options for patients as early as possible, and is expected to achieve strategies such as early stratified precision treatment. Beyond HER2‐positive breast cancer, a deep learning algorithm demonstrated clinical potential in TNBC through evaluating neoadjuvant therapy (NAT) response in 165 cases, achieving an AUC of 0.62 [50]. This predictive performance supports early treatment selection and stratified precision strategies for this aggressive subtype.

Immunotherapy targeting the programmed death‐1 (PD‐1) and programmed death‐ligand 1 (PD‐L1) pathway represents a promising emerging modality in cancer treatment. This approach activates anti‐tumor immune responses by inhibiting the PD‐1/PD‐L1 checkpoint interaction. In lung cancer, PD‐1/PD‐L1 inhibitors have demonstrated superior response rates and survival outcomes compared to chemotherapy in patients with tumors expressing PD‐L1 in over 50% of cells [88]. Similarly, in breast cancer, the KEYNOTE‐355 trial confirmed that for metastatic TNBC, pembrolizumab combined with chemotherapy confers enhanced clinical benefit in patients with PD‐L1‐positive tumors [89].

While the PD‐L1 staining process is time‐consuming and exhibits potential instability over time, PD‐L1 status prediction and subsequent immunotherapy efficacy assessment can be achieved through routinely available, stable H&E slides. Multiple studies have employed artificial intelligence models utilizing H&E specimens to overcome these limitations, demonstrating capabilities in predicting PD‐L1 staining patterns and evaluating implications for immunotherapy/neoadjuvant treatment outcomes [68, 69, 90]. For example, Gil Shamai et al. applied deep learning to PD‐L1 prediction in 3376 breast cancers, achieving AUCs of 0.91–0.93 [90]. Huang et al. leveraged AI to assist PD‐L1 and immune marker (CD8+, CD163+) assessments, finding integrated evaluations of these markers outperformed pathologist‐alone assessments for neoadjuvant response prediction [69].

### 3.4 Application of AI Models Based on Gene Expression Profile in NAT for Breast Cancer

Breast cancer is a heterogeneous disease presenting complex tumorigenic mechanisms and divergent NAT responses. Even cancers of identical molecular phenotypes may differ in treatment responses. Gene expression profiling and subsequent precision subtyping efforts individualize care by better defining beneficiary populations. Commercial genetic tests like Oncotype DX, MammaPrint, PAM50, and EndoPredict/Breast Cancer Index currently aid neoadjuvant screening. In a meta‐analysis of 1774 breast cancers, Boland correlated higher 21‐gene recurrence risks (> 25) with elevated pCR rates [91]. Likewise, 70‐gene MammaPrint testing associated increased pCR in HR+/HER2‐ breast cancers with high‐risk designation [92].

However, molecular subtype testing requires the extraction of patient blood or fresh tumor tissue, or tumor paraffin rolls to complete. Different times and types of samples have an impact on molecular subtype testing, leading to unstable test results or large deviations. Computational pathology offers advantages by rapidly deriving biomarkers solely from stable H&E slides. For example, Cho et al. [93] developed the Lunit SCOPE algorithm to identify 1343 breast cancers, predicting 21‐gene scores and histopathologic parameters for recurrence risk and neoadjuvant benefit stratification—validated in TCGA data. Patients with Oncotype DX scores > 25 demonstrated higher risk and therapy responsiveness.

Presently, AI models for breast cancer—constructed using histomorphological features from epithelium, stroma, TILs, molecular subtyping, or integrated genomic profiles—demonstrate potential for predicting neoadjuvant therapy efficacy and risk stratification. Despite demonstrating competitive performance across studies, these models face critical limitations: (1) most rely on small, single‐center retrospective datasets (e.g., Basavanhally et al.'s [53] cohort of *n* = 12), raising concerns about overfitting and generalizability; (2) low‐dimensional data constraints and lack of interpretability. Advancing oncology research now integrates multidimensional, multiscale ‘big data’ from molecular variations, histopathology, radiology, and clinical records.

### 3.5 Application of AI Technology Based on Multidimensional Data in NAT for Breast Cancer

**FIGURE 3:**
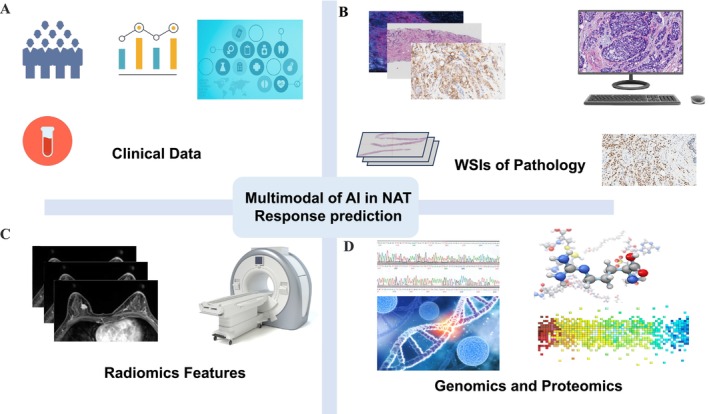
AI‐driven multimodal framework for breast cancer NAT response prediction, integrating clinical data (A), histopathological features (B), radiomics (C), and molecular profiling (genomics/proteomics) (D).

The future AI enables multimodal analysis capturing heterogeneity unappreciated by individual data. AI models integrate information from diverse sources/contexts to provide more accurate patient prediction information (Figure 3). This allows clinicians to formulate more targeted medical plans and achieve the goal of personalized medical care.

For example, Carrell et al.'s rule‐based natural language processing system combines pathology and clinical modules to predict breast cancer recurrence with an accuracy of 92% and a sensitivity of 96% [94]. It can replace manual annotation to some extent and reduce manual costs. Wu et al. developed a prognostic model in a multicenter study that synergistically integrates digital histopathological imaging with multimodal clinical data to predict recurrence risk in hormone receptor‐positive/human epidermal growth factor receptor 2‐negative (HR+/HER2‐) early‐stage breast cancer patients receiving adjuvant chemo‐endocrine therapy. The model demonstrated robust discriminative performance, achieving an AUC of 0.86 [95].

Histopathology and radiomics are integral to clinical strategy in breast cancer management. Digital pathology involves digitizing glass slides into virtual WSIs for macroscopic and microscopic examination. Compared to conventional methods, WSI provides advantages such as expedited retrieval, centralized data storage, remote collaboration, and reproducible, objective evaluations independent of interpretive variability. Similarly, medical imaging modalities like computed tomography (CT) and magnetic resonance imaging (MRI) generate 3D renderings of (pre)malignant breast lesions to augment microscopic findings. The integration of radiomic and histopathomic biomarkers may enhance prognostic accuracy and therapeutic response predictions.

In the study of Narui et al. combining pre‐NAT MRI findings with biopsy histopathology on H&E stained slides achieved 90.3% accuracy for predicting neoadjuvant response—exceeding single modality models [96]. Further research [97] also validated the integration of pathomics signatures with radiomics and clinical features, demonstrating significant potential in predicting NAT efficacy for breast cancer.

Breast cancer exhibits a multifaceted malignant cell ecological milieu, comprising a complex TME. The interactions among diverse components within this microenvironment significantly contribute to tumor initiation, progression, and treatment response.

However, comprehensively characterizing the tumor ecosystem poses financial and logistical challenges.

Research of Stephen‐John Sammut [70], they amalgamated 168 patients' pre‐therapy mutational profiling, proliferative activity, immune parameters, and T‐cell function using AI, achieving 0.87 AUC for pathological complete response risk modeling. It follows that neoadjuvant effectiveness correlates with multidimensional tumor‐related ecological characteristics. AI‐based models can integrate multidimensional data and employ machine learning techniques to capture the holistic ecological characteristics of tumors. By integrating tumor‐related information from various perspectives, it can accurately predict clinical treatment outcomes, offering dependable information to aid treatment decisions.

### 3.6 Different Patterns of Residual Disease for Breast Cancer and Its Application of AI

Emerging evidence highlights prognostic correlations between patterns of residual lesions in non‐pCR patients post‐neoadjuvant therapy and long‐term outcomes. Tinterri et al. [98] demonstrated in a 219‐patient cohort that scattered residual patterns correlate with improved overall survival (HR = 0.62, *p* = 0.03), while Pastorello et al. [74] established contraction patterns (central regression with peripheral satellites) as pCR predictors (OR = 3.14, *p* = 0.017) despite marginal residuals increasing recurrence risk (HR = 1.92, *p* = 0.042). In addition, residual pure intralymphatic carcinoma component only after NAT in breast cancer is also associated with poor outcome [99].

AI demonstrates robust predictive capacity in residual lesion characterization through multimodal data integration. Xu et al. [100] designed a pathology‐MRI fusion model (*n* = 586) achieving 0.92 AUC for dual prediction of pathological complete response status and residual lesion spatial topology. Complementing this, Huang et al. [101] developed a longitudinal MRI‐based machine learning framework (*n* = 432) exhibiting 0.985 diagnostic accuracy in identifying contraction patterns during tumor regression dynamics, offering intraoperative guidance for surgical margin determination.

## 4 Challenges and Prospects

### 4.1 Prospective Clinical Validation and Regulation

To overcome the limitations of retrospective studies, such as selection bias and data heterogeneity, more prospective studies with direct clinical relevance have been gradually undertaken in recent years. Mao's team innovatively constructed a multi‐center prediction model for breast cancer by integrating whole‐slide pathological images (WSI), multimodal imaging data, and clinical risk factors. This model exhibited excellent predictive performance in an independent prospective test set (AUC = 0.909), confirming the clinical value of multimodal data fusion strategies in improving prediction accuracy [71]. Despite these breakthroughs, prospective studies with strict multi‐center designs and standardized data collection processes are still relatively scarce at present. Large‐scale prospective validation is crucial for comprehensively verifying the stability, universality, and clinical practicability of AI methods in predicting NAT response for breast cancer patients.

Furthermore, insufficient regulatory oversight risks perpetuating algorithmic bias, diagnostic inaccuracies, or overreliance on automated systems. Despite the U.S. Food and Drug Administration (FDA) approval of select AI‐driven diagnostic tools for whole slide imaging (WSI) analysis [102], the establishment of robust regulatory frameworks remains imperative. These frameworks should incorporate dynamic quality control mechanisms encompassing algorithm transparency, data traceability, iterative model validation, and user compliance monitoring. In high‐stakes clinical decision‐making scenarios, mandatory independent third‐party validation and rigorous ethical oversight frameworks must be implemented to ensure equilibrium between technological innovation and safeguarding patient rights.

### 4.2 Standardization and Generalization

Furthermore, the standardization and generalizability of AI‐driven breast cancer prediction models face significant challenges due to data heterogeneity, class imbalance, technological variability, and clinical complexity. For instance, institutional disparities in pathological slide digitization protocols (e.g., variations in WSI scanner brands) and inconsistencies in feature extraction methodologies hinder the development of universally generalizable clinical models [103], often resulting in poor algorithmic adaptability. Compounding this issue is the current absence of large‐scale, high‐quality breast pathology image databases with standardized acquisition protocols. Most studies rely on limited, single‐institution datasets from homogeneous WSI sources, increasing risks of algorithmic overfitting [53]. To address these limitations, standardized guidelines must be established to ensure imaging data consistency across diverse patient demographics and scanning equipment. Additionally, collaborative efforts should prioritize creating a multi‐institutional, open‐access database and validation platforms with unified evaluation metrics and reporting frameworks to enhance reproducibility and clinical translatability.

### 4.3 Interpretability of AI Models

Despite demonstrating high predictive accuracy in breast cancer neoadjuvant therapy outcomes [104], the clinical translation of AI‐driven models remains impeded by their inherent “black box” nature. While post hoc interpretability techniques (e.g., SHAP, LIME) generate localized explanations via heatmaps or feature contribution scores, these methods often fail to establish clinically meaningful correlations with pathological or biological principles [105, 106]. For example, the AI model developed by Shao Zhimin et al., which predicts antibody‐drug conjugate (ADC) efficacy by integrating spatial topological features and immune microenvironment parameters, exhibits limited biological plausibility due to insufficient visualization of its multimodal fusion mechanism [61]. To address these limitations, future research should prioritize: integration of inherently interpretable architectures, development of clinician‐centric interfaces, and cross‐institutional validation of interpretation consistency to ensure model transparency across diverse patient cohorts and clinical workflows.

### 4.4 Large‐Scale Multimodal Dataset

Current research commonly integrates histopathological H&E of epithelial/tissue features, molecular characteristics, genomics, and clinical data within AI‐driven systems to identify patients most likely to benefit from NAT. Through predictive models, AI can automate laborious tasks, reduce clinician workload, and facilitate personalized treatment by assimilating heterogeneous information. Crucially, integrating diverse data modalities enhances predictive accuracy and robustness, providing clinicians with insights for model selection in clinical scenarios, as shown in HER2‐positive breast cancer management where AI synthesizes histopathological, radiomics, and binding site data to guide therapy [107]. Despite this, to construct a complete intelligent assisted treatment prediction system, it is still necessary to incorporate the full‐cycle clinical data of patients and achieve in‐depth collaboration in the diagnosis and treatment process. Future development should focus on overcoming challenges related to data harmonization, model interpretability, and clinical integration to realize the full potential of AI, drawing parallels to the promising yet carefully considered application of AI in integrating complex multidimensional data for managing conditions like geriatric syndromes [108].

## 5 Conclusion

In conclusion, the multifactorial complexity of NAT response prediction necessitates synergistic integration of artificial intelligence with clinical expertise. This demands interdisciplinary partnerships spanning academic institutions, industry stakeholders, and medical practitioners to: (1) curate multimodal repositories with standardized validation protocols; (2) address technical bottlenecks in predictive algorithm development; (3) implement robust AI‐driven clinical decision support systems for precise patient stratification and prognostic prediction; ultimately accelerating clinical translation while ensuring clinician‐patient trust through interpretable AI architectures.

## Author Contributions

**Juan Ji:** writing – original draft, validation. **Fanglei Duan:** data curation, methodology, visualization. **Qiong Liao:** formal analysis, validation. **Hao Wang:** investigation. **Shiwei Liu:** resources. **Yang Liu:** conceptualization, project administration, supervision. **Zongyao Huang:** conceptualization, project administration, supervision.

## Ethics Statement

The authors have nothing to report.

## Consent

The authors have nothing to report.

## Conflicts of Interest

The authors declare no conflicts of interest.

## Data Availability

The data that support the findings of this study are available from the corresponding author upon reasonable request.

## References

[1] A. N. Giaquinto , H. Sung , K. D. Miller , et al., “Breast Cancer Statistics, 2022,” CA: A Cancer Journal for Clinicians 72, no. 6 (2022): 524–541. 36190501 10.3322/caac.21754

[2] O. M. Filho , G. Viale , S. Stein , et al., “Impact of HER2 Heterogeneity on Treatment Response of Early‐Stage HER2‐Positive Breast Cancer: Phase II Neoadjuvant Clinical Trial of T‐DM1 Combined With Pertuzumab,” Cancer Discovery 11, no. 10 (2021): 2474–2487. 33941592 10.1158/2159-8290.CD-20-1557 PMC8598376

[3] G. P. Dowling , G. R. Daly , A. Hegarty , et al., “Predictive Value of Pretreatment Circulating Inflammatory Response Markers in the Neoadjuvant Treatment of Breast Cancer: Meta‐Analysis,” British Journal of Surgery 111, no. 5 (2024): znae132. 38801441 10.1093/bjs/znae132 PMC11129713

[4] A. Shmatko , N. Ghaffari Laleh , M. Gerstung , and J. N. Kather , “Artificial Intelligence in Histopathology: Enhancing Cancer Research and Clinical Oncology,” Nature Cancer 3, no. 9 (2022): 1026–1038. 36138135 10.1038/s43018-022-00436-4

[5] J. S. Reis‐Filho and J. N. Kather , “Overcoming the Challenges to Implementation of Artificial Intelligence in Pathology,” Journal of the National Cancer Institute 115, no. 6 (2023): 608–612. 36929936 10.1093/jnci/djad048 PMC10248832

[6] R. Bostrom , H. Sawyer , and W. Tolles , “Instrumentation for Automatically Prescreening Cytological Smears,” Proceedings of the IRE 47 (1959): 1895–1900.

[7] K. Onji , S. Yoshida , S. Tanaka , et al., “Quantitative Analysis of Colorectal Lesions Observed on Magnified Endoscopy Images,” Journal of Gastroenterology 46, no. 12 (2011): 1382–1390. 21918927 10.1007/s00535-011-0459-x

[8] H. Irshad , S. Jalali , L. Roux , et al., “Automated Mitosis Detection Using Texture, SIFT Features and HMAX Biologically Inspired Approach,” Journal of Pathology Informatics 4, no. S12 (2013). 10.4103/2153-3539.109870 PMC3678748 23766934

[9] Y. LeCun , B. Boser , J. S. Denker , et al., “Handwritten Digit Recognition With a Back‐Propagation Network,” in Advances in Neural Information Processing Systems, ed. D. Touretzky (Morgan‐Kaufmann, 1990).

[10] Y. Lecun , L. Bottou , Y. Bengio , and P. Hafner , “Gradient‐Based Learning Applied to Document Recognition,” Proceedings of the IEEE 86, no. 11 (1998): 2278–2324.

[11] S. Brockmoeller , A. Echle , N. Ghaffari Laleh , et al., “Deep Learning Identifies Inflamed Fat as a Risk Factor for Lymph Node Metastasis in Early Colorectal Cancer,” Journal of Pathology 256, no. 3 (2022): 269–281. 34738636 10.1002/path.5831

[12] E. Wulczyn , D. F. Steiner , M. Moran , et al., “Interpretable Survival Prediction for Colorectal Cancer Using Deep Learning,” NPJ Digital Medicine 4, no. 1 (2021): 71. 33875798 10.1038/s41746-021-00427-2 PMC8055695

[13] C. Saillard , B. Schmauch , O. Laifa , et al., “Predicting Survival After Hepatocellular Carcinoma Resection Using Deep Learning on Histological Slides,” Hepatology 72, no. 6 (2020): 2000–2013. 32108950 10.1002/hep.31207

[14] O. J. Skrede , S. De Raedt , A. Kleppe , et al., “Deep Learning for Prediction of Colorectal Cancer Outcome: A Discovery and Validation Study,” Lancet 395, no. 10221 (2020): 350–360. 32007170 10.1016/S0140-6736(19)32998-8

[15] P. Mobadersany , S. Yousefi , M. Amgad , et al., “Predicting Cancer Outcomes From Histology and Genomics Using Convolutional Networks,” Proceedings of the National Academy of Sciences of the United States of America 115, no. 13 (2018): E2970–E2979. 29531073 10.1073/pnas.1717139115 PMC5879673

[16] J. Calderaro and J. N. Kather , “Artificial Intelligence‐Based Pathology for Gastrointestinal and Hepatobiliary Cancers,” Gut 70, no. 6 (2021): 1183–1193. 33214163 10.1136/gutjnl-2020-322880

[17] N. Coudray , P. S. Ocampo , T. Sakellaropoulos , et al., “Classification and Mutation Prediction From Non‐Small Cell Lung Cancer Histopathology Images Using Deep Learning,” Nature Medicine 24, no. 10 (2018): 1559–1567. 10.1038/s41591-018-0177-5 PMC9847512 30224757

[18] J. W. Hughes , N. Yuan , B. He , et al., “Deep Learning Evaluation of Biomarkers From Echocardiogram Videos,” eBioMedicine 73 (2021): 103613. 34656880 10.1016/j.ebiom.2021.103613 PMC8524103

[19] J. Chen , X. Chen , T. Li , L. Wang , and G. Lin , “Identification of Chromatin Organization‐Related Gene Signature for Hepatocellular Carcinoma Prognosis and Predicting Immunotherapy Response,” International Immunopharmacology 109 (2022): 108866. 35691273 10.1016/j.intimp.2022.108866

[20] A. Paul and D. P. Mukherjee , “Mitosis Detection for Invasive Breast Cancer Grading in Histopathological Images,” IEEE Transactions on Image Processing 24, no. 11 (2015): 4041–4054. 26219094 10.1109/TIP.2015.2460455

[21] N. Wahab , A. Khan , and Y. S. Lee , “Two‐Phase Deep Convolutional Neural Network for Reducing Class Skewness in Histopathological Images Based Breast Cancer Detection,” Computers in Biology and Medicine 85 (2017): 86–97. 28477446 10.1016/j.compbiomed.2017.04.012

[22] J. Xu , L. Xiang , Q. Liu , et al., “Stacked Sparse Autoencoder (SSAE) for Nuclei Detection on Breast Cancer Histopathology Images,” IEEE Transactions on Medical Imaging 35, no. 1 (2016): 119–130. 26208307 10.1109/TMI.2015.2458702 PMC4729702

[23] M. Veta , P. J. van Diest , and J. P. W. Pluim , “Cutting Out the Middleman: Measuring Nuclear Area in Histopathology Slides Without Segmentation,” in Medical Image Computing and Computer‐Assisted Intervention – MICCAI 2016 (Springer International Publishing, 2016), 632–639.

[24] F. Xing , Y. Xie , and L. Yang , “An Automatic Learning‐Based Framework for Robust Nucleus Segmentation,” IEEE Transactions on Medical Imaging 35, no. 2 (2016): 550–566. 26415167 10.1109/TMI.2015.2481436

[25] M. Veta , P. J. van Diest , S. M. Willems , et al., “Assessment of Algorithms for Mitosis Detection in Breast Cancer Histopathology Images,” Medical Image Analysis 20, no. 1 (2015): 237–248. 25547073 10.1016/j.media.2014.11.010

[26] M. Amgad , L. A. Atteya , H. Hussein , et al., “NuCLS: A Scalable Crowdsourcing Approach and Dataset for Nucleus Classification and Segmentation in Breast Cancer,” GigaScience 11 (2022): giac037. 35579553 10.1093/gigascience/giac037 PMC9112766

[27] R. Nateghi , H. Danyali , and M. S. Helfroush , “A Deep Learning Approach for Mitosis Detection: Application in Tumor Proliferation Prediction From Whole Slide Images,” Artificial Intelligence in Medicine 114 (2021): 102048. 33875159 10.1016/j.artmed.2021.102048

[28] D. Romo‐Bucheli , A. Janowczyk , H. Gilmore , E. Romero , and A. Madabhushi , “Automated Tubule Nuclei Quantification and Correlation With Oncotype DX Risk Categories in ER+ Breast Cancer Whole Slide Images,” Scientific Reports 6 (2016): 32706. 27599752 10.1038/srep32706 PMC5013328

[29] A. Janowczyk and A. Madabhushi , “Deep Learning for Digital Pathology Image Analysis: A Comprehensive Tutorial With Selected Use Cases,” Journal of Pathology Informatics 7 (2016): 29. 27563488 10.4103/2153-3539.186902 PMC4977982

[30] C. Dang , Z. Qi , T. Xu , et al., “Deep Learning‐Powered Whole Slide Image Analysis in Cancer Pathology,” Laboratory Investigation, (2025). 10.1016/j.labinv.2025.104186 40306572

[31] C. Boissin , Y. Wang , A. Sharma , et al., “Deep Learning‐Based Risk Stratification of Preoperative Breast Biopsies Using Digital Whole Slide Images,” Breast Cancer Research 26, no. 1 (2024): 90. 38831336 10.1186/s13058-024-01840-7 PMC11145850

[32] J. Luo , B. Diao , J. Wang , et al., “A Deep‐Learning‐Based Clinical Risk Stratification for Overall Survival in Adolescent and Young Adult Women With Breast Cancer,” Journal of Cancer Research and Clinical Oncology 149, no. 12 (2023): 10423–10433. 37277578 10.1007/s00432-023-04955-0 PMC11798295

[33] C. Y. Huang , R. F. Chang , C. Y. Lin , et al., “Deep‐Learning Model to Improve Histological Grading and Predict Upstaging of Atypical Ductal Hyperplasia / Ductal Carcinoma In Situ on Breast Biopsy,” Histopathology 84, no. 6 (2024): 983–1002. 38288642 10.1111/his.15144

[34] R. Albusayli , J. D. Graham , N. Pathmanathan , et al., “Artificial Intelligence‐Based Digital Scores of Stromal Tumour‐Infiltrating Lymphocytes and Tumour‐Associated Stroma Predict Disease‐Specific Survival in Triple‐Negative Breast Cancer,” Journal of Pathology 260, no. 1 (2023): 32–42. 36705810 10.1002/path.6061

[35] R. K. Mondol , E. K. A. Millar , A. Sowmya , and E. Meijering , “BioFusionNet: Deep Learning‐Based Survival Risk Stratification in ER+ Breast Cancer Through Multifeature and Multimodal Data Fusion,” IEEE Journal of Biomedical and Health Informatics 28, no. 9 (2024): 5290–5302. 38913518 10.1109/JBHI.2024.3418341

[36] P. H. Tan , I. Ellis , K. Allison , et al., “The 2019 World Health Organization Classification of Tumours of the Breast,” Histopathology 77, no. 2 (2020): 181–185. 32056259 10.1111/his.14091

[37] C. Mercan , M. Balkenhol , R. Salgado , et al., “Deep Learning for Fully‐Automated Nuclear Pleomorphism Scoring in Breast Cancer,” NPJ Breast Cancer 8, no. 1 (2022): 120. 36347887 10.1038/s41523-022-00488-w PMC9643392

[38] C. Genestie , B. Zafrani , B. Asselain , et al., “Comparison of the Prognostic Value of Scarff‐Bloom‐Richardson and Nottingham Histological Grades in a Series of 825 Cases of Breast Cancer: Major Importance of the Mitotic Count as a Component of Both Grading Systems,” Anticancer Research 18, no. 1B (1998): 571–576. 9568179

[39] L. Roux , D. Racoceanu , N. Loménie , et al., “Mitosis Detection in Breast Cancer Histological Images an ICPR 2012 Contest,” Journal of Pathology Informatics 4 (2013): 8. 23858383 10.4103/2153-3539.112693 PMC3709417

[40] J. S. Meyer , C. Alvarez , C. Milikowski , et al., “Breast Carcinoma Malignancy Grading by Bloom‐Richardson System vs Proliferation Index: Reproducibility of Grade and Advantages of Proliferation Index,” Modern Pathology 18, no. 8 (2005): 1067–1078. 15920556 10.1038/modpathol.3800388

[41] C. van Dooijeweert , P. J. van Diest , S. M. Willems , et al., “Significant Inter‐ and Intra‐Laboratory Variation in Grading of Invasive Breast Cancer: A Nationwide Study of 33,043 Patients in The Netherlands,” International Journal of Cancer 146, no. 3 (2020): 769–780. 30977119 10.1002/ijc.32330 PMC6916412

[42] Y. Wang , B. Acs , S. Robertson , et al., “Improved Breast Cancer Histological Grading Using Deep Learning,” Annals of Oncology 33, no. 1 (2022): 89–98. 34756513 10.1016/j.annonc.2021.09.007

[43] A. Sharma , S. K. Lövgren , K. L. Eriksson , et al., “Validation of an AI‐Based Solution for Breast Cancer Risk Stratification Using Routine Digital Histopathology Images,” Breast Cancer Research 26, no. 1 (2024): 123. 39143539 10.1186/s13058-024-01879-6 PMC11323658

[44] C. Y. Xu , J. W. Xie , C. X. Yang , Y. N. Jiang , Z. H. Zhang , and J. Xu , “Prediction of Response to Neoadjuvant Chemotherapy for Breast Cancer Based on Histomorphology Analysis of Needle Biopsy Images,” Sichuan Da Xue Xue Bao. Yi Xue Ban 52, no. 2 (2021): 279–285. 33829703 10.12182/20210360505 PMC10408908

[45] H. Zeng , S. Qiu , S. Zhuang , et al., “Deep Learning‐Based Predictive Model for Pathological Complete Response to Neoadjuvant Chemotherapy in Breast Cancer From Biopsy Pathological Images: A Multicenter Study,” Frontiers in Physiology 15 (2024): 1279982. 38357498 10.3389/fphys.2024.1279982 PMC10864440

[46] T. B. Fisher , G. Saini , T. S. Rekha , et al., “Digital Image Analysis and Machine Learning‐Assisted Prediction of Neoadjuvant Chemotherapy Response in Triple‐Negative Breast Cancer,” Breast Cancer Research 26, no. 1 (2024): 12. 38238771 10.1186/s13058-023-01752-y PMC10797728

[47] F. Li , Y. Yang , Y. Wei , et al., “Deep Learning‐Based Predictive Biomarker of Pathological Complete Response to Neoadjuvant Chemotherapy From Histological Images in Breast Cancer,” Journal of Translational Medicine 19, no. 1 (2021): 348. 34399795 10.1186/s12967-021-03020-z PMC8365907

[48] K. Saednia , W. T. Tran , and A. Sadeghi‐Naini , “A Hierarchical Self‐Attention‐Guided Deep Learning Framework to Predict Breast Cancer Response to Chemotherapy Using Pre‐Treatment Tumor Biopsies,” Medical Physics 50, no. 12 (2023): 7852–7864. 37403567 10.1002/mp.16574

[49] K. Saednia , A. Lagree , M. A. Alera , et al., “Quantitative Digital Histopathology and Machine Learning to Predict Pathological Complete Response to Chemotherapy in Breast Cancer Patients Using Pre‐Treatment Tumor Biopsies,” Scientific Reports 12, no. 1 (2022): 9690. 35690630 10.1038/s41598-022-13917-4 PMC9188550

[50] S. Krishnamurthy , P. Jain , D. Tripathy , et al., “Predicting Response of Triple‐Negative Breast Cancer to Neoadjuvant Chemotherapy Using a Deep Convolutional Neural Network‐Based Artificial Intelligence Tool,” JCO Clinical Cancer Informatics 7 (2023): e2200181. 36961981 10.1200/CCI.22.00181 PMC10530970

[51] F. Li , Y. Yang , Y. Wei , et al., “Predicting Neoadjuvant Chemotherapy Benefit Using Deep Learning From Stromal Histology in Breast Cancer,” NPJ Breast Cancer 8, no. 1 (2022): 124. 36418332 10.1038/s41523-022-00491-1 PMC9684483

[52] S. M. Hacking , J. Karam , K. Singh , et al., “Whole Slide Image Features Predict Pathologic Complete Response and Poor Clinical Outcomes in Triple‐Negative Breast Cancer,” Pathology, Research and Practice 246 (2023): 154476. 37146413 10.1016/j.prp.2023.154476

[53] A. N. Basavanhally , S. Ganesan , S. Agner , et al., “Computerized Image‐Based Detection and Grading of Lymphocytic Infiltration in HER2+ Breast Cancer Histopathology,” IEEE Transactions on Biomedical Engineering 57, no. 3 (2010): 642–653. 19884074 10.1109/TBME.2009.2035305

[54] J. Saltz , R. Gupta , L. Hou , et al., “Spatial Organization and Molecular Correlation of Tumor‐Infiltrating Lymphocytes Using Deep Learning on Pathology Images,” Cell Reports 23, no. 1 (2018): 181–193.e7. 29617659 10.1016/j.celrep.2018.03.086 PMC5943714

[55] M. Amgad , A. Sarkar , C. Srinivas , et al., “Joint Region and Nucleus Segmentation for Characterization of Tumor Infiltrating Lymphocytes in Breast Cancer,” Proceedings of SPIE–The International Society for Optical Engineering 10956 (2019): 109560M. 10.1117/12.2512892 PMC6988758 31997849

[56] Z. Lu , S. Xu , W. Shao , et al., “Deep‐Learning‐Based Characterization of Tumor‐Infiltrating Lymphocytes in Breast Cancers From Histopathology Images and Multiomics Data,” JCO Clinical Cancer Informatics 4 (2020): 480–490. 32453636 10.1200/CCI.19.00126 PMC7265782

[57] F. Negahbani , R. Sabzi , B. Pakniyat Jahromi , et al., “PathoNet Introduced as a Deep Neural Network Backend for Evaluation of Ki‐67 and Tumor‐Infiltrating Lymphocytes in Breast Cancer,” Scientific Reports 11, no. 1 (2021): 8489. 33875676 10.1038/s41598-021-86912-w PMC8055887

[58] H. Le , R. Gupta , L. Hou , et al., “Utilizing Automated Breast Cancer Detection to Identify Spatial Distributions of Tumor‐Infiltrating Lymphocytes in Invasive Breast Cancer,” American Journal of Pathology 190, no. 7 (2020): 1491–1504. 32277893 10.1016/j.ajpath.2020.03.012 PMC7369575

[59] S. Makhlouf , N. Wahab , M. Toss , et al., “Evaluation of Tumour Infiltrating Lymphocytes in Luminal Breast Cancer Using Artificial Intelligence,” British Journal of Cancer 129, no. 11 (2023): 1747–1758. 37777578 10.1038/s41416-023-02451-3 PMC10667537

[60] K. A. Fanucci , Y. Bai , V. Pelekanou , et al., “Image Analysis‐Based Tumor Infiltrating Lymphocytes Measurement Predicts Breast Cancer Pathologic Complete Response in SWOG S0800 Neoadjuvant Chemotherapy Trial,” NPJ Breast Cancer 9, no. 1 (2023): 38. 37179362 10.1038/s41523-023-00535-0 PMC10182981

[61] D. Ma , L. J. Dai , X. R. Wu , et al., “Spatial Determinants of Antibody‐Drug Conjugate SHR‐A1811 Efficacy in Neoadjuvant Treatment for HER2‐Positive Breast Cancer,” Cancer Cell 43, no. 6 (2025): 1061–1075.e7. 40215979 10.1016/j.ccell.2025.03.017

[62] J. Ogier du Terrail , A. Leopold , C. Joly , et al., “Federated Learning for Predicting Histological Response to Neoadjuvant Chemotherapy in Triple‐Negative Breast Cancer,” Nature Medicine 29, no. 1 (2023): 135–146. 10.1038/s41591-022-02155-w 36658418

[63] W. Aswolinskiy , E. Munari , H. M. Horlings , et al., “PROACTING: Predicting Pathological Complete Response to Neoadjuvant Chemotherapy in Breast Cancer From Routine Diagnostic Histopathology Biopsies With Deep Learning,” Breast Cancer Research 25, no. 1 (2023): 142. 37957667 10.1186/s13058-023-01726-0 PMC10644597

[64] B. Shen , A. Saito , A. Ueda , et al., “Development of Multiple AI Pipelines That Predict Neoadjuvant Chemotherapy Response of Breast Cancer Using H&E‐Stained Tissues,” Journal of Pathology. Clinical Research 9, no. 3 (2023): 182–194. 36896856 10.1002/cjp2.314 PMC10073928

[65] B. Li , F. Li , Z. Liu , et al., “Deep Learning With Biopsy Whole Slide Images for Pretreatment Prediction of Pathological Complete Response to Neoadjuvant Chemotherapy in Breast Cancer: A Multicenter Study,” Breast 66 (2022): 183–190. 36308926 10.1016/j.breast.2022.10.004 PMC9619175

[66] G. Shamai , Y. Binenbaum , R. Slossberg , I. Duek , Z. Gil , and R. Kimmel , “Artificial Intelligence Algorithms to Assess Hormonal Status From Tissue Microarrays in Patients With Breast Cancer [Published Correction Appears in JAMA Network Open. 2019 Aug 2;2(8):e1911247],” JAMA Network Open 2, no. 7 (2019): e197700. 31348505 10.1001/jamanetworkopen.2019.7700 PMC6661721

[67] S. Farahmand , A. I. Fernandez , F. S. Ahmed , et al., “Deep Learning Trained on Hematoxylin and Eosin Tumor Region of Interest Predicts HER2 Status and Trastuzumab Treatment Response in HER2+ Breast Cancer,” Modern Pathology 35, no. 1 (2022): 44–51. 34493825 10.1038/s41379-021-00911-w PMC10221954

[68] X. Wang , L. Wang , H. Bu , et al., “How Can Artificial Intelligence Models Assist PD‐L1 Expression Scoring in Breast Cancer: Results of Multi‐Institutional Ring Studies,” NPJ Breast Cancer 7, no. 1 (2021): 61. 34039982 10.1038/s41523-021-00268-y PMC8155065

[69] Z. Huang , W. Shao , Z. Han , et al., “Cancer Neoadjuvant Chemotherapy Responses From Multi‐Stain Histopathologic Images,” NPJ Precision Oncology 7, no. 1 (2023): 14. 36707660 10.1038/s41698-023-00352-5 PMC9883475

[70] S. J. Sammut , M. Crispin‐Ortuzar , S. F. Chin , et al., “Multi‐Omic Machine Learning Predictor of Breast Cancer Therapy Response,” Nature 601, no. 7894 (2022): 623–629. 34875674 10.1038/s41586-021-04278-5 PMC8791834

[71] N. Mao , Y. Dai , H. Zhou , et al., “A Multimodal and Fully Automated System for Prediction of Pathological Complete Response to Neoadjuvant Chemotherapy in Breast Cancer,” Science Advances 11, no. 18 (2025): eadr1576. 40305609 10.1126/sciadv.adr1576 PMC12042891

[72] S. Liu , E. Mou , S. Zeng , et al., “Therapeutic Effect of Trastuzumab in Neoadjuvant‐Treated HER2‐Positive Breast Cancer With Low Infiltrating Level of Tumor‐Infiltrating Lymphocytes,” Cancer Management and Research 12 (2020): 3145–3153. 32440212 10.2147/CMAR.S248071 PMC7221413

[73] P. M. Collins , M. J. Brennan , J. A. Elliott , et al., “Neoadjuvant Chemotherapy for Luminal a Breast Cancer: Factors Predictive of Histopathologic Response and Oncologic Outcome,” American Journal of Surgery 222 (2021): 368–376. 33334569 10.1016/j.amjsurg.2020.11.053

[74] R. G. Pastorello , A. Laws , S. Grossmith , et al., “Clinico‐Pathologic Predictors of Patterns of Residual Disease Following Neoadjuvant Chemotherapy for Breast Cancer,” Modern Pathology 34, no. 5 (2021): 875–882. 33219297 10.1038/s41379-020-00714-5

[75] H. Duanmu , S. Bhattarai , H. Li , et al., “A Spatial Attention Guided Deep Learning System for Prediction of Pathological Complete Response Using Breast Cancer Histopathology Images,” Bioinformatics 38, no. 19 (2022): 4605–4612. 35962988 10.1093/bioinformatics/btac558 PMC9525016

[76] S. C. Hagenaars , S. de Groot , D. Cohen , et al., “Tumor‐Stroma Ratio Is Associated With Miller‐Payne Score and Pathological Response to Neoadjuvant Chemotherapy in HER2‐Negative Early Breast Cancer,” International Journal of Cancer 149, no. 5 (2021): 1181–1188. 34043821 10.1002/ijc.33700 PMC8362217

[77] Y. Wang , A. S. Brodsky , J. Xiong , M. L. Lopresti , D. Yang , and M. B. Resnick , “Stromal Clusterin Expression Predicts Therapeutic Response to Neoadjuvant Chemotherapy in Triple Negative Breast Cancer,” Clinical Breast Cancer 18, no. 3 (2018): e373–e379. 28890185 10.1016/j.clbc.2017.08.007

[78] F. Li , H. Chen , X. Lu , et al., “Combining the Tumor‐Stroma Ratio With Tumor‐Infiltrating Lymphocytes Improves the Prediction of Pathological Complete Response in Breast Cancer Patients,” Breast Cancer Research and Treatment 202, no. 1 (2023): 173–183. 37528265 10.1007/s10549-023-07026-7

[79] N. M. Atallah , N. Wahab , M. S. Toss , et al., “Deciphering the Morphology of Tumor‐Stromal Features in Invasive Breast Cancer Using Artificial Intelligence,” Modern Pathology 36, no. 10 (2023): 100254. 37380057 10.1016/j.modpat.2023.100254

[80] J. Zhao , M. Liu , C. Zhu , et al., “Cancer‐Associated Fibroblasts and Metabolic Reprogramming Predict Pathologic Response to Neoadjuvant PD‐1 Blockade in Resected Non‐Small Cell Lung Cancer,” Cellular Oncology 48, no. 4 (2025): 1105–1119. 40358847 10.1007/s13402-025-01067-4 PMC12238122

[81] F. Miglietta , M. Ragazzi , B. Fernandes , et al., “A Prognostic Model Based on Residual Cancer Burden and Tumor‐Infiltrating Lymphocytes on Residual Disease After Neoadjuvant Therapy in HER2+ Breast Cancer,” Clinical Cancer Research 29, no. 17 (2023): 3429–3437. 37417941 10.1158/1078-0432.CCR-23-0480 PMC10472099

[82] A. D. Lopes , N. A. L. Galdino , A. B. Figueiredo , et al., “Systemic Immune Mediators Reflect Tumour‐Infiltrating Lymphocyte Intensity and Predict Therapeutic Response in Triple‐Negative Breast Cancer,” Immunology 169, no. 2 (2023): 229–241. 36703241 10.1111/imm.13627

[83] P. W. Whitworth , P. D. Beitsch , J. V. Pellicane , et al., “Distinct Neoadjuvant Chemotherapy Response and 5‐Year Outcome in Patients With Estrogen Receptor‐Positive, Human Epidermal Growth Factor Receptor 2‐Negative Breast Tumors That Reclassify as Basal‐Type by the 80‐Gene Signature,” JCO Precision Oncology 6, no. 1 (2022): e2100463. 35476550 10.1200/PO.21.00463 PMC9200401

[84] L. Wang , J. R. Asirvatham , Y. Ma , E. S. Reisenbichler , and J. M. Jorns , “HER‐2/Neu‐Positive Breast Cancer Neoadjuvant Chemotherapy Response After Implementation of 2018 ASCO/CAP Focused Update,” Breast Journal 27, no. 8 (2021): 631–637. 34018281 10.1111/tbj.14241

[85] V. Guarneri , M. V. Dieci , G. Bisagni , et al., “De‐Escalated Therapy for HR+/HER2+ Breast Cancer Patients With Ki67 Response After 2‐Week Letrozole: Results of the PerELISA Neoadjuvant Study,” Annals of Oncology 30, no. 6 (2019): 921–926. 30778520 10.1093/annonc/mdz055 PMC6594455

[86] Y. Chen , H. Li , A. Janowczyk , et al., “Computational Pathology Improves Risk Stratification of a Multi‐Gene Assay for Early Stage ER+ Breast Cancer,” NPJ Breast Cancer 9, no. 1 (2023): 40. 37198173 10.1038/s41523-023-00545-y PMC10192429

[87] N. Naik , A. Madani , A. Esteva , et al., “Deep Learning‐Enabled Breast Cancer Hormonal Receptor Status Determination From Base‐Level H&E Stains,” Nature Communications 11, no. 1 (2020): 5727. 10.1038/s41467-020-19334-3 PMC7670411 33199723

[88] J. E. Chaft , A. Rimner , W. Weder , C. G. Azzoli , M. G. Kris , and T. Cascone , “Evolution of Systemic Therapy for Stages I‐III Non‐Metastatic Non‐Small‐Cell Lung Cancer,” Nature Reviews. Clinical Oncology 18, no. 9 (2021): 547–557. 10.1038/s41571-021-00501-4 PMC9447511 33911215

[89] J. Cortes , D. W. Cescon , H. S. Rugo , et al., “Pembrolizumab Plus Chemotherapy Versus Placebo Plus Chemotherapy for Previously Untreated Locally Recurrent Inoperable or Metastatic Triple‐Negative Breast Cancer (KEYNOTE‐355): A Randomised, Placebo‐Controlled, Double‐Blind, Phase 3 Clinical Trial,” Lancet 396, no. 10265 (2020): 1817–1828. 33278935 10.1016/S0140-6736(20)32531-9

[90] G. Shamai , A. Livne , A. Polónia , et al., “Deep Learning‐Based Image Analysis Predicts PD‐L1 Status From H&E‐Stained Histopathology Images in Breast Cancer,” Nature Communications 13, no. 1 (2022): 6753. 10.1038/s41467-022-34275-9 PMC9643479 36347854

[91] M. R. Boland , A. Al‐Maksoud , É. J. Ryan , et al., “Value of a 21‐Gene Expression Assay on Core Biopsy to Predict Neoadjuvant Chemotherapy Response in Breast Cancer: Systematic Review and Meta‐Analysis,” British Journal of Surgery 108, no. 1 (2021): 24–31. 33640948 10.1093/bjs/znaa048

[92] S. B. Vliek , F. S. Hilbers , A. Jager , et al., “Ten‐Year Follow‐Up of the Observational RASTER Study, Prospective Evaluation of the 70‐Gene Signature in ER‐Positive, HER2‐Negative, Node‐Negative, Early Breast Cancer,” European Journal of Cancer 175 (2022): 169–179. 36126477 10.1016/j.ejca.2022.07.036

[93] S. Y. Cho , J. H. Lee , J. M. Ryu , et al., “Deep Learning From HE Slides Predicts the Clinical Benefit From Adjuvant Chemotherapy in Hormone Receptor‐Positive Breast Cancer Patients,” Scientific Reports 11, no. 1 (2021): 21043. 34671078 10.1038/s41598-021-00546-6 PMC8528879

[94] D. S. Carrell , S. Halgrim , D. T. Tran , et al., “Using Natural Language Processing to Improve Efficiency of Manual Chart Abstraction in Research: The Case of Breast Cancer Recurrence,” American Journal of Epidemiology 179, no. 6 (2014): 749–758. 24488511 10.1093/aje/kwt441 PMC3939853

[95] X. Wu , Y. Li , J. Chen , et al., “Multimodal Recurrence Risk Prediction Model for HR+/HER2‐ Early Breast Cancer Following Adjuvant Chemo‐Endocrine Therapy: Integrating Pathology Image and Clinicalpathological Features,” Breast Cancer Research 27, no. 1 (2025): 27. 40148997 10.1186/s13058-025-01968-0 PMC11951786

[96] K. Narui , T. Ishikawa , M. S. Oba , et al., “Prediction of Pathological Complete Response After Neoadjuvant Chemotherapy in Breast Cancer by Combining Magnetic Resonance Imaging and Core Needle Biopsy,” Surgical Oncology 35 (2020): 447–452. 33045629 10.1016/j.suronc.2020.10.002

[97] X. Li and F. Yan , “Predictive Value of Background Parenchymal Enhancement on Breast Magnetic Resonance Imaging for Pathological Tumor Response to Neoadjuvant Chemotherapy in Breast Cancers: A Systematic Review,” Cancer Imaging 24, no. 1 (2024): 35. 38462607 10.1186/s40644-024-00672-0 PMC10926651

[98] C. Tinterri , B. Fernandes , A. Zambelli , et al., “The Impact of Different Patterns of Residual Disease on Long‐Term Oncological Outcomes in Breast Cancer Patients Treated With Neo‐Adjuvant Chemotherapy,” Cancers 16, no. 2 (2024): 376. 38254865 10.3390/cancers16020376 PMC10814808

[99] H. Lee , Y. Jang , Y. A. Cho , and E. Y. Cho , “Residual Pure Intralymphatic Carcinoma Component Only (Lymphovascular Tumor Emboli Without Invasive Carcinoma) After Neoadjuvant Chemotherapy Is Associated With Poor Outcome: Not Pathologic Complete Response,” Human Pathology 145 (2024): 1–8. 38311186 10.1016/j.humpath.2024.02.002

[100] N. Xu , X. Guo , Z. Ouyang , et al., “Multiparametric MRI‐Based Radiomics Combined With Pathomics Features for Prediction of the Efficacy of Neoadjuvant Chemotherapy in Breast Cancer,” Heliyon 10, no. 2 (2024): e24371. 38298695 10.1016/j.heliyon.2024.e24371 PMC10827766

[101] Y. Huang , W. Chen , X. Zhang , et al., “Prediction of Tumor Shrinkage Pattern to Neoadjuvant Chemotherapy Using a Multiparametric MRI‐Based Machine Learning Model in Patients With Breast Cancer,” Frontiers in Bioengineering and Biotechnology 9 (2021): 662749. 34295877 10.3389/fbioe.2021.662749 PMC8291046

[102] S. Mukhopadhyay , M. D. Feldman , E. Abels , et al., “Whole Slide Imaging Versus Microscopy for Primary Diagnosis in Surgical Pathology: A Multicenter Blinded Randomized Noninferiority Study of 1992 Cases (Pivotal Study),” American Journal of Surgical Pathology 42, no. 1 (2018): 39–52. 28961557 10.1097/PAS.0000000000000948 PMC5737464

[103] G. Verghese , J. K. Lennerz , D. Ruta , et al., “Computational Pathology in Cancer Diagnosis, Prognosis, and Prediction ‐ Present Day and Prospects,” Journal of Pathology 260, no. 5 (2023): 551–563. 37580849 10.1002/path.6163 PMC10785705

[104] J. Guo , B. Chen , H. Cao , et al., “Cross‐Modal Deep Learning Model for Predicting Pathologic Complete Response to Neoadjuvant Chemotherapy in Breast Cancer,” NPJ Precision Oncology 8, no. 1 (2024): 189. 39237596 10.1038/s41698-024-00678-8 PMC11377584

[105] S. Z. Vahed , S. M. H. Khatibi , Y. R. Saadat , et al., “Introducing Effective Genes in Lymph Node Metastasis of Breast Cancer Patients Using SHAP Values Based on the mRNA Expression Data,” PLoS One 19, no. 8 (2024): e0308531. 39150915 10.1371/journal.pone.0308531 PMC11329117

[106] T. Alelyani , M. M. Alshammari , A. Almuhanna , and O. Asan , “Explainable Artificial Intelligence in Quantifying Breast Cancer Factors: Saudi Arabia Context,” Healthcare (Basel) 12, no. 10 (2024): 1025. 38786433 10.3390/healthcare12101025 PMC11120946

[107] X. Deng , Y. Yan , Z. Zhan , et al., “A Glimpse Into the Future: Integrating Artificial Intelligence for Precision HER2‐Positive Breast Cancer Management,” iMetaOmics 1 (2024): e19. 10.1002/imo2.19 PMC12806260 41675540

[108] L. Zhang and J. Li , “Prospects for the Application of Artificial Intelligence in Geriatrics,” Journal of Translational Internal Medicine 12, no. 6 (2025): 531–533, 10.1515/jtim-2024-0034. 39802448 PMC11720927

